# A Modified Enucleation and Deflation to Treat Large Ameloblastoma in the Mandible

**DOI:** 10.1155/2023/8835892

**Published:** 2023-07-05

**Authors:** Andi Setiawan Budihardja, Bakhrul Lutfianto

**Affiliations:** ^1^Department of Oral and Maxillofacial Surgery, Faculty of Medicine, University of Pelita Harapan, Siloam Hospital Lippo Village, Tangerang, Indonesia; ^2^Department of Oral Maxillofacial Surgery, Faculty of Dentistry, University of Muhammadiah, Yogyakarta, Indonesia

## Abstract

**Objective:**

Treatment of large ameloblastoma with a conservative method. *Case Presentation.* Ameloblastomas are benign tumors of odontogenic origin. These tumors are locally invasive with a high recurrence rate. This case report will present a conservative approach to treating large ameloblastoma in the mandible using modified enucleation and deflation technique. The patient presents with a recurrence of large unicystic ameloblastoma. The following patient is treated with the two-stage enucleation and deflation technique. One-year post-treatment follow-up shows satisfactory healing, with good bone regeneration in the defected area, showing no signs of recurrence.

**Conclusion:**

The technique that will be discussed in this case report provides an alternative treatment for ameloblastomas of the mandible. This approach can avoid radical operative procedures, such as jaw resection and bone reconstruction.

## 1. Introduction

Ameloblastomas are one of the most common benign tumors of the jaw. These tumors are known to be of odontogenic origin. Although benign, ameloblastomas are often locally invasive and can destroy the jaw. If inadequately treated, recurrence rates are very high [1].

Ameloblastoma tumors are classified into unicystic, multicystic, peripheral, and malignant. Malignant ameloblastomas, such as ameloblastic carcinomas, are very rarely found [2].

The current gold standard of treatment for ameloblastomas is radical surgery—involving jaw resection with a safety margin of 1 cm, and then resecting the soft tissue structures affected. Primary bone reconstruction is mandatory for large tumors with extensive bone damage.

Bone reconstruction is performed using autogenous avascular bone grafts (if the defect is less than 1 cm). With larger defects, reconstruction must be done using a microvascular bone transplant.

Although bone reconstruction surgery using the microvascular technique is available in several countries and medical centers as part of a routine medical procedure, in other parts of the world and centers, bone reconstruction with the microvascular approach is a challenge in itself. There remains an insufficient number of surgeons with skills in this field, as well as a lack of tools and technology. Reconstruction surgery is also very high cost and not affordable for many patients in more developing parts of the world.

The treatment of ameloblastoma using the dredging method is done by step-by-step deflation and enucleation. With this method, jaw bone resection can be avoided.

## 2. Case Presentation

A 40-year-old male patient was admitted to our Department of Oral and Maxillofacial Surgery, with the primary complaint of swelling in his right mandible 6 months ago. Over 6 months, he reports that the swelling has gotten bigger causing pain and difficulty in opening his mouth.

From the initial history, it was found that the patient had undergone surgery 3 years ago at another hospital. The procedure done was curettage, and histological findings revealed a multicystic ameloblastoma.

**Figure 1:**
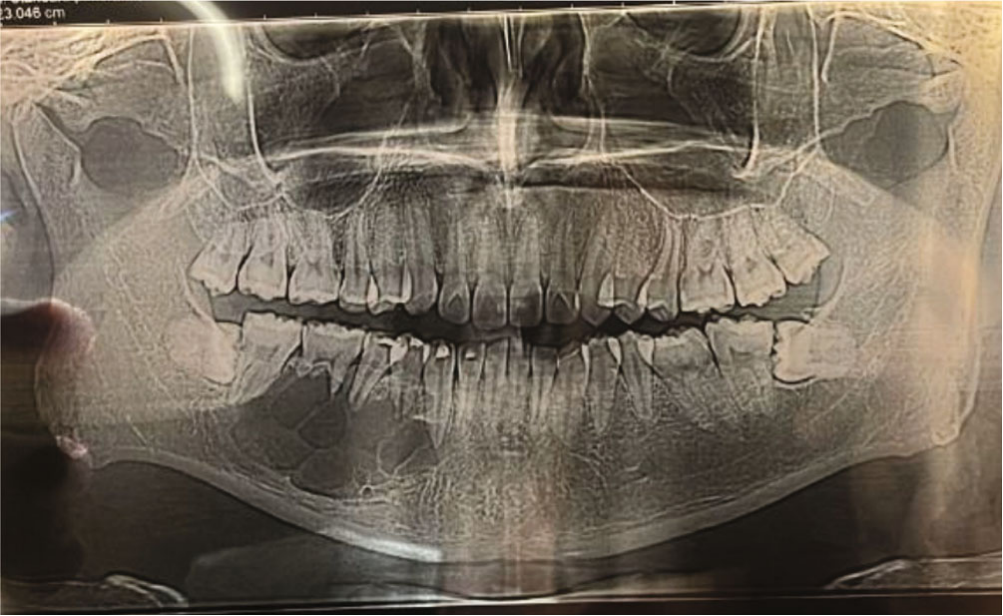
Multicystic ameloblastoma in the right mandible.

**Figure 2:**
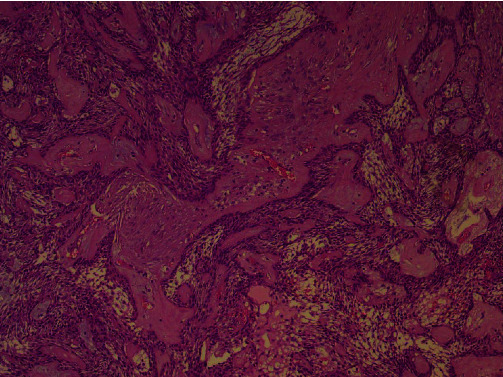
Histological examination from the soft tissue mass showed multicystic ameloblastoma.

Orthopantomogram (OPG) (Figure 1) and computed tomography revealed a large multicystic mass on the right mandible that started in the tooth #43 region up to the angle of the mandible. The condyle region was not affected. The next step for this patient was an incisional biopsy from the soft tissue tumor—the results revealed a multicystic ameloblastoma (Figure 2).

**Figure 3:**
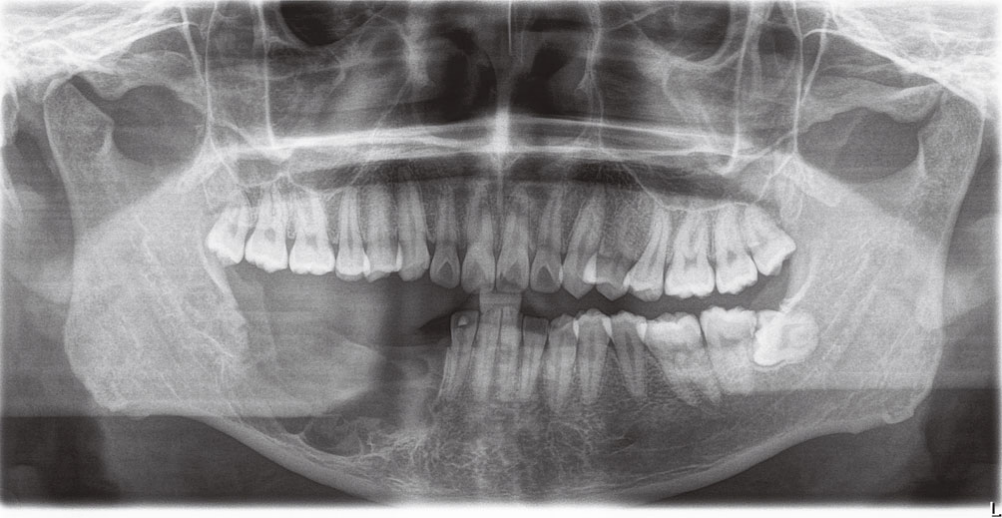
Post-operative OPG after enucleation and deflation.

After a discussion with the patient and his family, it was decided to approach treatment using the dredging method. The first surgery was performed using the dredging method, in which enucleation and deflation of the tumor were done under general anaesthesia (Figure 3). The wound was left open for secondary healing. A gauze with antibiotic ointment was put into the open cavity. The patient is scheduled for a follow-up every 3 days to irrigate the wound with sodium chloride and chlorhexidine solution, curettage of the new tissue on the basal area of the bone, and followed a gauze change.

In the second week, a double impression was made of the lower jaw to create an acrylic obturator prosthesis, with the acrylic plate intended to cover the area of the defect.

**Figure 4:**
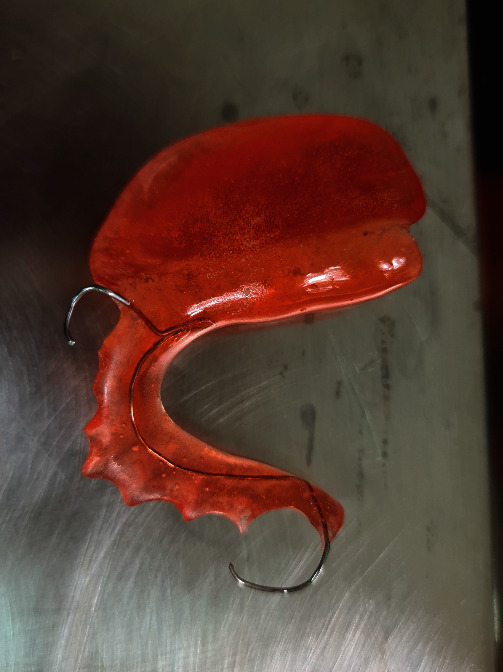
Obturator prosthesis.

When the obturator prosthesis (Figure 4) was ready, the gauze filling was removed, and the patient was instructed to use the acrylic prosthesis for 24 hours. The patient is then asked to follow up within 10 days. If the prosthesis was difficult to insert, the acrylic was ground on the area that compresses the new and growing granulation tissue.

The second surgery was done 3 months after the first surgery. This surgery included deflation and enucleation followed by a histopathological examination of the new tissue formation from the affected area. Histopathological findings revealed no signs of tumor growth. The obturator prosthesis is further used for another 6 months.

**Figure 5:**
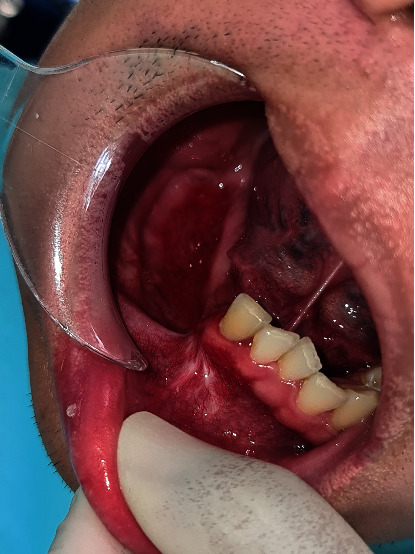
Intraoral situation after the second enucleation, and deflation showed new tissue formation.

In the sixth month (Figure 5), another deflation and enucleation surgery is performed along with pathological analysis of the soft tissue. The findings did not show signs of tumor. The patient is then asked to follow up every 4 weeks to control the use of the obturator prosthesis.

**Figure 6:**
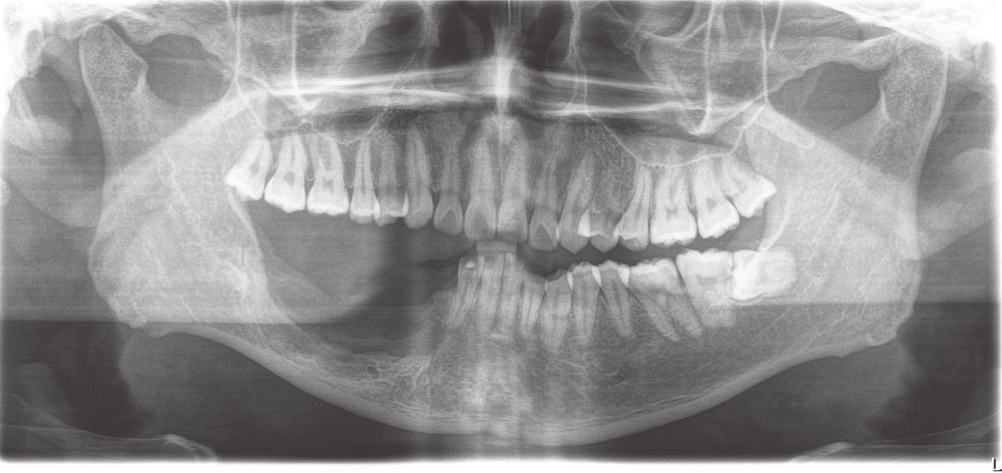
OPG 1 year after initial operation showing good new bone formation and no recurrence.

One year after the first surgery, OPG (Figure 6) reveals new bone formation on the defected area with no signs of recurrent tumor growth.

## 3. Discussion

Although ameloblastomas are benign tumors of odontogenic origin, these tumors are known to be invasive and destructive with high recurrence rates. A study by Lau and Samman states that in a systematic review, the recurrence rate for unicystic ameloblastomas is 3.6% for resection, 30.5% for enucleation, 16% for enucleation by application of Carnoy's solution, and 18% for marsupialization [1].

Hendra et al. showed in a meta-analytic study that the rate of recurrence for multicystic ameloblastoma is 8% with radical treatment and 41% with conservative treatment, whereas, for unicystic ameloblastoma, the recurrence rates are 2–31% [3].

Au et al. and Sasaki et al. revealed that the 5-, 10-, and 15-year recurrence rates for ameloblastoma were 9.3%, 17.6%, and 24.4% [4, 5].

The gold standard treatment for ameloblastomas with extensive bone destruction is mandibular resection surgery in conjunction with primary defect reconstruction done using microvascular bone transplantation. However, this treatment is inaccessible in many countries due to high costs and limitations with doctors specializing in the field, as well as inadequate medical facilities.

The treatment of ameloblastoma using the dredging method was demonstrated and published by Kawamura et al. A combination of enucleation and deflation is done for unicystic ameloblastomas. Enucleation methods can be done up to five times until tumor cells are no longer found on histopathological examination [6, 7].

In the case study above, a modified version of the dredging method was performed—after initial enucleation and deflation, the tumor area was left open and filled with gauze. Then, enucleation was performed under local anesthesia once per week for three consecutive months. The affected tissue was removed, allowing for the formation of new bone growth in the mandible. The prosthetic acrylic obturator allowed the area to be easily irrigated from leftover food debris and ensured the area remained open.

In the third month, a histologic test was performed to ensure that no remaining tumor growth was found. The same was done in the sixth month. If two histopathological findings in a row did not show tumor growth, new bone and tissue formation were allowed by progressively sanding the base of the prosthesis to allow new bone and soft tissue to grow.

A one-year follow-up for this case revealed healthy new bone growth with no signs of a recurring tumor. This treatment may be considered as an alternative to more extensive and invasive surgery.

## 4. Conclusion

The modified dredging method, as a conservative surgical treatment, may represent a reliable approach for the management of ameloblastoma. This procedure can eliminate tumor cell growth in the scar tissue and accelerate new bone formation. In addition, this method can eliminate the need for radical surgeries and complex bone reconstruction.

## Data Availability

Data supporting this research article are available from the corresponding author or first author upon reasonable request.
